# Prognostic Significance of PNI in Patients With Pancreatic Head Cancer Undergoing Laparoscopic Pancreaticoduodenectomy

**DOI:** 10.3389/fsurg.2022.897033

**Published:** 2022-06-01

**Authors:** Peng Jiang, Xiaocheng Li, Shupeng Wang, Yahui Liu

**Affiliations:** Department of Hepatobiliary and Pancreatic Surgery, First Hospital of Jilin University, Changchun, China

**Keywords:** pancreatic head cancer, laparoscopic pancreaticoduodenectomy, prognosis nutritional index, overall survival, morbidity & mortality

## Abstract

**Background:**

Recently, several prognosis indicators based on inflammatory and nutritional factors, such as the neutrophil-to-lymphocyte ratio (NLR), plated-to-lymphocyte (PLR), lymphocyte-to-monocyte (LMR) and prognosis nutritional index (PNI), have been proposed as prognosis factors for several cancers. However, few studies have looked into PNI. The goal of this research was to see if preoperative PNI had any predictive value in patients with pancreatic head cancer who were having a laparoscopic pancreaticoduodenectomy.

**Methods:**

From February 11, 2018 to May 31, 2019, two hundred and fifty-one pancreatic head carcinoma patients were retrospectively enrolled. The receiver operator characteristic (ROC) curve was used to determine the cut-off value. Patients were divided into two groups: PNI > 45.1 (high PNI group) and PNI < 45.1 (low PNI group), and clinic-pathological data was compared between the two groups. The link between PNI and NLR, PLR, and LMR, and their effect on overall survival. In addition, the factors of postoperative survival were analyzed univariate and multivariate.

**Results:**

PNI, NLR, PLR and LMR cut-off values were 45.1, 3.7,287.2 and 3.6, respectively. Between the two groups of patients, the low PNI group exhibited considerably higher PLR and lower LMR. PNI had a negative correlation with PLR and NLR (*r* = −0.329, *p* < 0.001 and *r* = 0.170, *p* = 0.014), but a positive correlation with LMR (*r* = 0.476, *p* < 0.001). The high PNI group had a considerably greater survival rate than the low PNI group (median survival days, 217 vs. 468, log-rank = 45.92, *p *< 0.001). PNI < 45.1(HR: 0.357, 95 percent CI, 0.263–0.485, *p* < 0.001) and LMR <3.6(HR: 0.705, 95 percent CI, 0.528–0.942, *p* < 0.018) were revealed to be possible predictive variable in univariate analysis. Only PNI <45.1 was found to be an independent predictive factor in multivariate analysis (HR: 0.359, 95%CI,: 0.256–0.502, *p* < 0.001).

**Conclusions:**

Our findings shoe that PNI is linked to a variety of systemic inflammatory response and can be used to predict survival in individuals with pancreatic head cancer.

## Introduction

Pancreatic cancer is the world’s third-leading cause of cancer death (1). Patients with pancreatic head cancer have a dismal prognosis as well (Figure 3A). The primary curative treatment for pancreatic head neoplasms is laparoscopic pancreaticoduodenectomy (LPD), which is a complicated surgical surgery (2). Perioperative morbidity and death rates for LPD have decreased over the last 20 years, and are estimated to be around 30%–40% and 3%, respectively (3). Even while surgical techniques and postoperative care are improving, there is always potential for improvement.

Many approaches based on inflammation and nutrition scores, such as the neutrophil-to-lymphocyte ratio (NLR), lymphocyte-to-monocyte (LMR), platelet-to-lymphocyte ratio (PLR), and prognostic nutritional index (PNI), have been used to the prognosis of pancreatic cancer in recent years (4–9). Poor nutritional status raises the risk of infection, delays wound healing, reduces coagulation function and makes the vascular wall more brittle, all of which increase the risk of postoperative problems (10). It can encourage tumor growth by reducing tumor immunity (11). The effects of systemic inflammation on tumor proliferation and survival, angiogenesis, metastasis, and treatment responsiveness have all been linked to poor clinical outcomes (12).

Smale et al. presented the notion of prognostic nutrition index (PNI) for the time in 1981 (13). The PNI concept used in this work was proposed by Onodera et al. in 1984 and was computed using serum albumin concentration and peripheral blood lymphocyte count. It was first used to predict preoperative risk factors and surgical indications for colorectal cancer patients, but it is now commonly utilized as a nutritional evaluation parameter for cancer patients (14). The laparoscopic pancreaticoduodenectomy is a difficult procedure with a high rate of morbidity and mortality. Prognosis prediction is useful for deciding the time of an operation and providing perioperative management guidelines.

To present, no study has looked the link between PNI and survival in patients with pancreatic head cancer who have had LPD. The primary goal of this study was to determine the predictive significance of PNI in patients with pancreatic head cancer who were undergoing LPD and to establish a link between PNI and systemic inflammatory response.

## Materials and Methods

### Patients

From February 11, 2018 to May 31, 2019, 207 pancreatic head carcinoma patients were retrospectively gathered from the Department of Hepatobiliary and Pancreatic Surgery, First Hospital of Jilin University. For pancratia cancer, the American Joint Committee on Cancer 8th edition tumor-node-metastasis (TNM) staging system was used for clinical and pathological staging (15). The following criteria were used to determine who was eligible: (1) 18–75 years old. (2) The diagnosis of pancreatic head carcinoma was clear and LPD treatment was performed (Total mesopancras excision (TMpE) was performed in all patients). (3) Complete clinicopathological records and follow-up records are available. The following criteria were used to determine who was ineligible (1) The existence of additional lesions in the malignant tumor, or the primary invasion of portal vein, hepatic artery, hepatic vein and inferior vena cava, lymph nodes or distant metastasis. (2) Preoperative chemotherapy, immunotherapy and other anti-tumor treatments were performed. (3) Parenteral nutrition was performed 2 weeks before surgery. (4) There are other inflammatory diseases caused by pancreatic head cancer. Totally, 44 patients were excluded and 207 were enrolled in the study. The majority postoperative follow-up was done over the telephone and as an outpatient. The longest follow-up duration was 2,059 days, and the median follow-up length was 947 days, with the longest follow-up period finishing on December 8, 2021. Survival time was computed from the date of surgery to the time of final follow-up or the date of death. All of the enrolled patients’ clinical case information was comprehensive.

### Preoperative and Postoperative Information

These perioperative data were included in the study: gender, Preoperative biliary drainage, American Society of Anesthesiologists (ASA) score, age, body mass index (BMI), preoperative carbohydrate antigen 19-9 (CA19-9), preoperative carbohydrate antigen 125 (CA125) in each group. The Clavien–Dindo classification was used to classify postoperative problems (16). White blood cell (WBC) count, neutrophil count, lymphocyte count, monocyte count, platelet count and serum albumin were all taken 3 days before to surgery. The ratios of neutrophil to lymphocyte (NLR), platelet to lymphocyte (PLR) and lymphocyte to monocyte (LMR) were computed. Serum albumin (g/L) + 0.005 * total lymphocyte count * 10^9^/L was used to determine the PNI. The number of lymph nodes obtained, the number of positive lymph nodes, hospitalization days, inpatient expenses, R0 resection rate and pathological test results all included in the postoperative data.

### Statistical Analysis

The Chi-square test and Fisher’s exact probability test were used to compare categorical variables between groups. The median and range of continuous data were calculated and compared using the Mann–Whitney U test. The minimal *p*-value on receiver operating characteristic (ROC) curve is used to establish the cut-off value. The Kaplan–Meier technique was used to compute overall survival (OS), which was then compared using the log-rank test. For both univariate and multivariate analyses, COX regression was utilized. The Cox proportional hazards regression model was used in IBM SPSS Statistical for windows, version 22.0, for both univariate and multivariate analyses (IBM Corp, USA). Statistically significant was defined as a *p* value of less than 0.05.

## Results

### PNI, NLR, PLR and LMR Cut-Off Values

**Figure 1:**
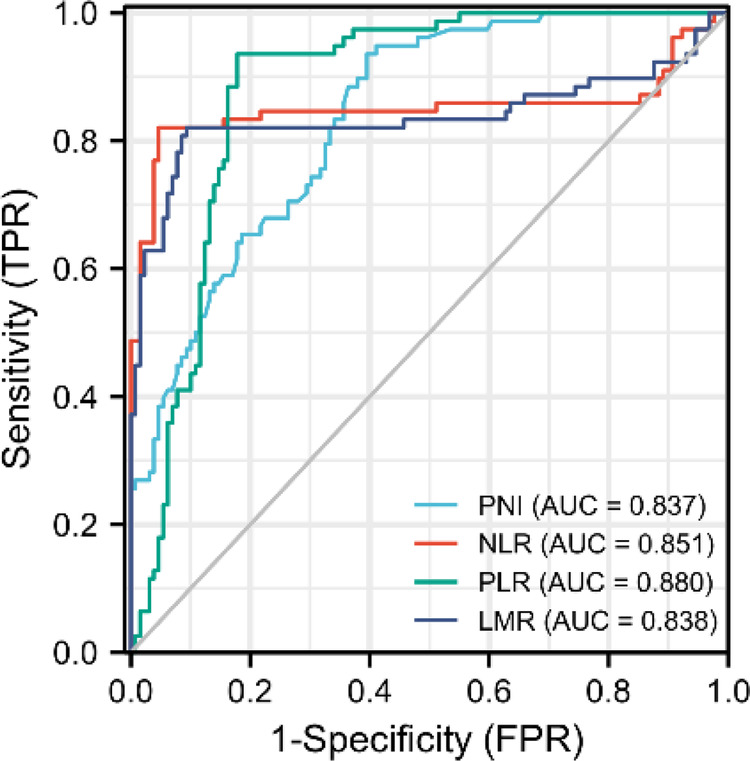
Roc curves of PNI, NLR, PLR and LMR. PNI, prognostic nutritional index; NLR, neutrophil-to-lymphocyte ratio; PLR, platelet-to-lymphocyte ratio; LMR, lymphocyte-to-monocyte.

PNI had a mean value of 46.9 ± 5.8 in 207 patients, with a minimum value of 34.8, and the maximum value of 64.0. The PNI ROC curve was drawn using 1-year survival rate, and the area under the curve was 0.837. The Youden index had the highest sensitivity and specificity when the PNI was 45.1, with a sensitivity of 93% and specificity of 61%. As a result, the high PNI group was defined as PNI > 45.1, while the low PNI group was established as PNI < 45.1. NLR, PLR and LMR Cut-off values were chosen in the same manner, and were 3.7, 287.1 and 3.6, respectively, which were similar to early studies (10, 17–22) (Figure 1).

### Comparison Between PNI < 45.1 and PNI > 45.1

The study included 207 patients, 83 of whom in the PNI < 45.1 group and 124 were in the PNI > 45.1 group. There was no significant difference in the number of problems of Clavien-Dindo grade (≥III) between the two groups of patients, however the low PNI group had significantly longer hospital stays and higher hospital costs. Furthermore, there were also statistical variations in PLR and LMR between the patient groups. It is worth mentioning that total mesopancras excision (TMpE) was performed in all patients undergoing laparoscopic pancreaticoduodenectomy to improve R0 removal rate, and there was no statistical difference between the two groups (84.3% vs. 89.5%, *p* = 0.271). Table 1 summarizes clinicopathological aspects in detail.

**Table 1:** Clinic-pathological features of the patients according to the PNI.

Characteristic	PNI < 45.1	PNI > 45.1	*p*
*N*	83	124	
Gender, *n* (%)			0.981
Female	34 (16.4%)	51 (24.6%)	
Male	49 (23.7%)	73 (35.3%)	
Preoperative biliary drainage, *n* (%)			0.579
No	65 (31.4%)	101 (48.8%)	
Yes	18 (8.7%)	23 (11.1%)	
ASA, *n* (%)			0.226
I	6 (2.9%)	7 (3.4%)	
II	62 (30%)	104 (50.2%)	
III	15 (7.2%)	12 (5.8%)	
IV	0 (0%)	1 (0.5%)	
Age, median (IQR)	61 (54, 67)	59 (51, 67)	0.353
BMI, median (IQR)	22.49 (20.92, 24.31)	22.77 (21.1, 24.4)	0.848
Preoperative CA199, U/mL, median (IQR)	116.53 (22.43, 281.4)	65.76 (16.87, 260.43)	0.365
Preoperative CA125, U/mL, median (IQR)	12.4 (9.07, 20.55)	12.97 (8.77, 18.21)	0.985
Perineural infiltration, *n* (%)			0.843
No	31 (15%)	48 (22.2%)	
Yes	52 (25.1%)	76 (36.7%)	
Vascular infiltration, *n* (%)			0.147
No	39 (18.8%)	71 (34.3%)	
Yes	44 (21.3%)	53 (25.6%)	
incisor involvement, *n* (%)			
No	73 (35.3%)	101 (48.8%)	0.211
Yes	10 (4.8%)	23 (11.1%)	
Clavien-Dindo grade (≥III)	9 (10.8%)	10 (8.1%)	0.497
NLR, median (IQR)	3.71 (2.52, 4.79)	3.79 (2.88, 6.38)	0.071
PLR, median (IQR)	233 (202.5, 266.13)	204.5 (185, 230.73)	<0.001
LMR, median (IQR)	2.76 (2.01, 3.52)	4 (3.04, 5.61)	<0.001
Lymph nodes obtained, median (IQR)	12 (9.5, 15)	11 (9, 15.25)	0.780
Positive lymph nodes, median (IQR)	1 (0, 2)	0 (0, 2)	0.471
Hospitalization days, median (IQR)	20 (16.5, 25)	19 (15, 23)	0.037
Hospitalization expenses, ¥, median (IQR)	134,489.41 (110,513.55, 159,715.62)	120,424.73 (108,646.24, 138,851.18)	0.025
R0 resection rate, *n* (%)	70 (84.3%)	111 (89.5%)	0.271
Pathological examination results, *n* (%)			0.931
Pancreatic ductal adenocarcinoma	52 (25.1%)	80 (38.6%)	
Intraductal papillary mucinous neoplasm (IPMN)	5 (2.4%)	10 (4.8%)	
Solid pseudopapillary tumor of the pancreas	9 (4.3%)	11 (5.3%)	
Pancreatic neuroendocrine tumor	2 (1%)	4 (1.9%)	
Others	15 (7.2%)	19 (9.2%)	

### Correlation Between PNI and Marks of the Systemic Inflammatory Response

**Figure 2:**
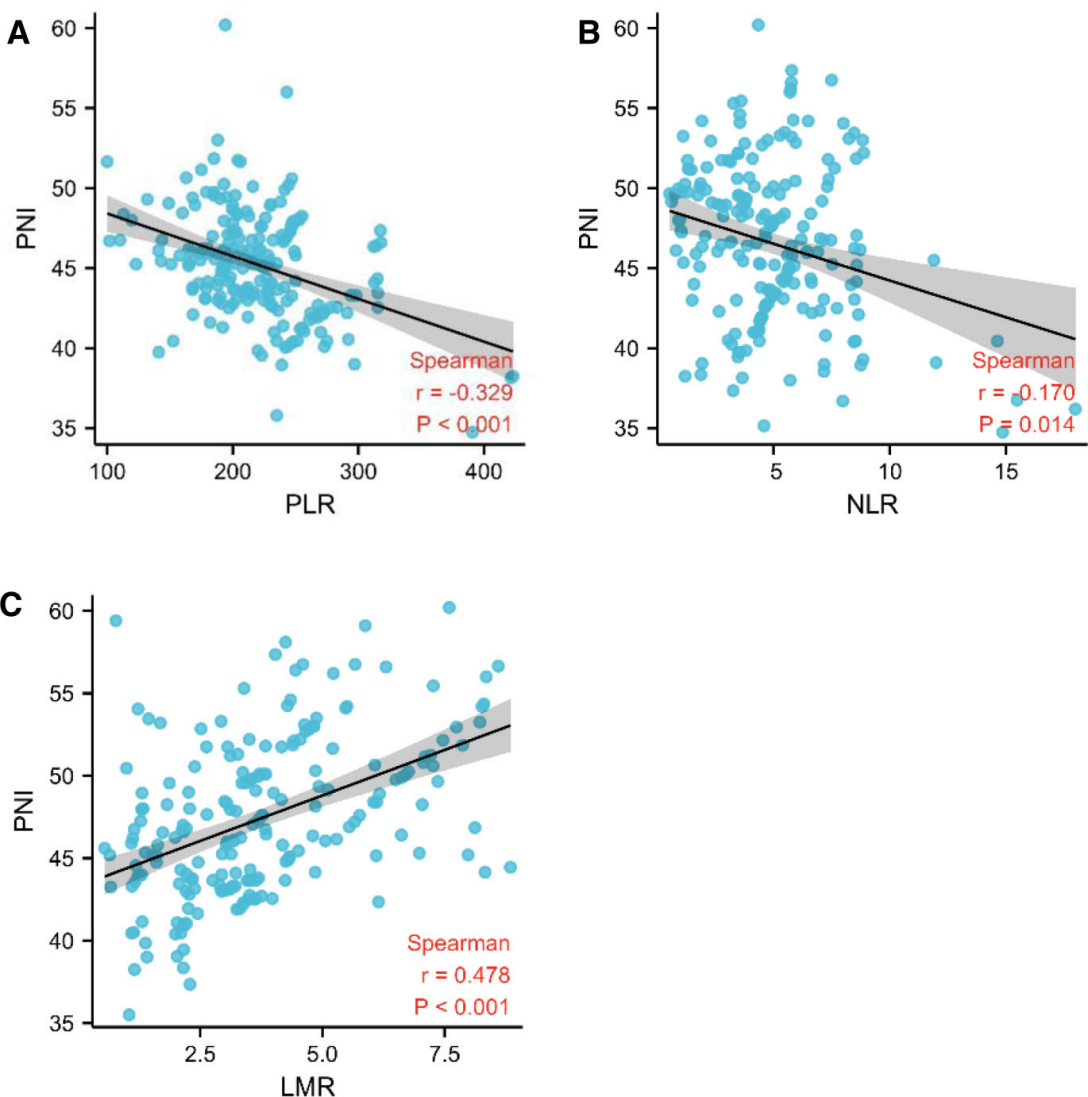
PNI was linked to the systemic inflammatory responsive marks. (**A**–**C**) Association between PNI and PLR, NLR, and LMR using Spearman’s correlation.

PNI is highly linked to systemic inflammatory markers such as LMR, NLR and PLR in patients with pancreatic head cancer, which has well reported in hematology. PNI had a negative correlation with PLR and NLR (*r* = −0.329, *p* < 0.001 and *r* = 0.170, *p* = 0.014), but a positive correlation with LMR (*r* = 0.476, *p* < 0.001). As a result, our finding show that PNI is linked to systemic inflammatory marks in pancreatic head carcinoma (Figure 2).

### PNI, LMR, NLR and PLR Were All Linked to OS in Patients with Pancreatic Head Carcinoma

**Figure 3:**
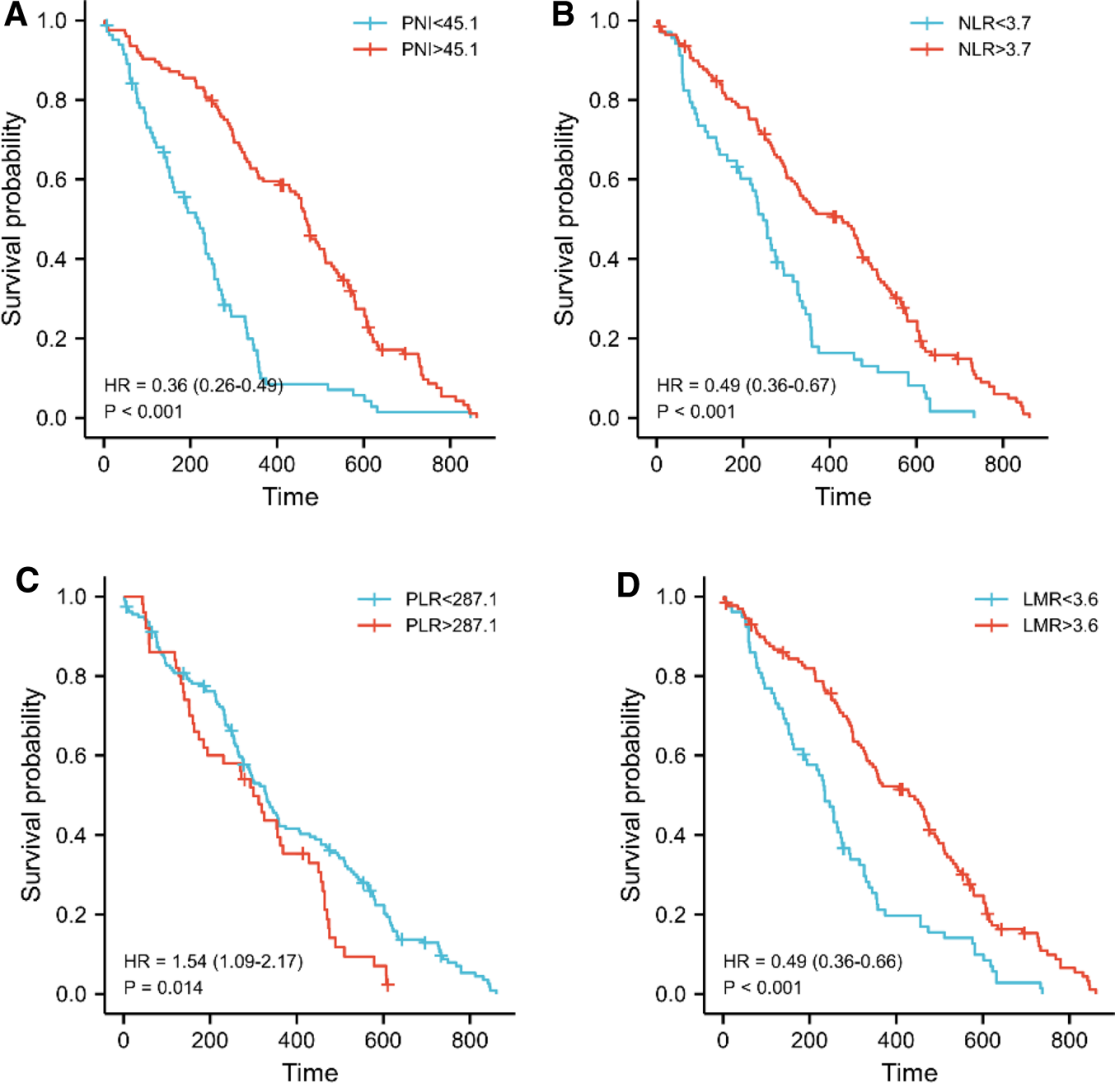
PNI was linked to overall survival in pancreatic head carcinoma patients. (**A**–**D**) Kaplan-Meier survival curve for all patients. The median values of PNI, LMR, NLR and PLR adopted the previous cutoff values. The *p*-value was calculated by the log-rank test.

First, we looked the link between PNI and OS, and Kaplan-Meier analysis revealed that the high PNI group outperformed the low PNI group (median survival days, 217 vs. 468, log-rank = 45.92, *p *< 0.001, Figure 3A). We continued to research the link between PLR, NLR, LMR and OS by using same method because of the substantial correlation between PNI and PLR, NLR, and LMR. The low NLR group had better survival than the high NLR group (median survival days, 428 vs. 246, log-rank = 20.85, *p *< 0.001, Figure 3B), and the low PLR group had better survival than the high PLR group (median survival days, 330 vs. 300, log-rank = 6.08, *p *= 0.014, Figure 3C), but the low LMR group had worse survival than the high LMR group (median survival days, 235 vs. 439, log-rank = 22.56, *p *< 0.001, Figure 3D),

### PNI is an Independent Predictor Factor for OS

PNI <45.1(HR: 0.357, 95%CI, 0.263–0.485, *p* < 0.001) and LMR <3.6(HR: 0.705, 95%CI, 0.528–0.942, *p* < 0.018) were discovered to be potential predictive factors based on the comparison of the low and high PNI groups in Table 1. Only PNI <45.1 was found to be an independent predictive factor in multivariate analysis (HR: 0.359, 95%CI, 0.256–0.502, *p* < 0.001). Specific information is presented in Table 2.

**Table 2:** PNI and overall survival in pancreatic head carcinoma patients: univariate and multivariate Cox regression analysis.

Characteristics	Total (*N*)	Univariate analysis		Multivariate analysis	
		Hazard ratio (95% CI)	*p* value	Hazard ratio (95% CI)	*p* value
PNI (<45.1 vs. >45.1)	83/124	0.357 (0.263–0.485)	<0.001	0.359 (0.256–0.502)	<0.001
NLR (<3.7 vs. >3.7)	99/108	1.061 (0.796–1.414)	0.686		
PLR (<287.1 vs. >287.1)	121/86	1.352 (0.795–2.298)	0.265		
LMR (<3.6 vs. >3.6)	113/94	0.705 (0.528–0.942)	0.018	0.957 (0.697–1.312)	0.783
Age (<60 vs. >60)	116/91	1.122 (0.841–1.498)	0.434		
Gender (Male vs. Female)	100/107	0.815 (0.612–1.086)	0.163		
Preoperative CA199, U/mL, (<100 vs. >100)	106/101	0.892 (0.669–1.189)	0.435		
Preoperative CA125, U/mL, (<12 vs. >12)	95/112	0.762 (0.571–1.015)	0.063	0.664 (0.492–0.894)	0.057
BMI (<22 vs. >22)	79/128	1.284 (0.952–1.732)	0.102		
Preoperative biliary drainage (Yes/No)	41/166	1.251 (0.871–1.797)	0.226		
ASA	207				
I	12	Reference			
II	167	1.229 (0.670–2.255)	0.505	1.104 (0.598–2.039)	0.752
III	27	2.024 (0.984–4.163)	0.055	1.496 (0.712–3.144)	0.288
IV	1	0.516 (0.067–3.994)	0.526	0.483 (0.062–3.790)	0.489
Perineural infiltration (T/F)	128/79	0.897 (0.661–1.217)	0.484		
Vascular infiltration (T/F)	97/110	0.749 (0.556–1.009)	0.058	0.864 (0.628–1.188)	0.368

## Discussion

PNI was demonstrated to be an independent predictor of overall survival in patients with pancreatic head cancer who underwent LPD surgery in this study. Low PNI values were also found to be substantially linked to a bad prognosis. This is the first research of preoperative PNI and postoperative overall survival in pancreatic head cancer that we are aware of. Kanda et al. looked at the link between PNI and overall survival in pancreatic cancer patients, but their study included all sites of pancreatic cancer (19). Different surgical procedures are required for pancreatic tumors at different locations, and the removal of some neighboring organs and blood arteries varies substantially. All these factors influence a patient’s prognosis and overall survival rate (23).As a result, this research is limited to pancreatic head cancer.

There is no consensus on the boundary between PNI, LMR, NLR and PLR values at this time. According to certain sources, PNI should be 47.3, 45, 40, 44.7, 46 and 46.8, LMR should be 3.33 and 3.4, NLR should be 2.75, 2.5, 2.65, 3 and 3.7, PLR should be 126, 200 and 247 (4, 6, 8, 10, 17–20, 24–28). The optimal cutoff values of PNI, LMR, NLR and PLR, according to sensitivity and specificity, were 45.1, 3.6, 3.7 and 287.1 respectively, in this investigation, which has good clinical practicability. The calculation of PNI cut-off value is crucial for determining preoperative nutritional status and whether preoperative nutritional therapy is required in patients with pancreatic head cancer. Preoperative nutrition, according to Harimoto et al., reduces the incidence rate of pancreatic fistula in skeletal muscle (SM) loss patients and improves the surgical outcomes in patients having pancreaticoduodenectomy (29). A more general conclusion, on the other hand, has to be confirmed by a large number of multicenter trials.

Malnutrition is a prevalent and dangerous concern with pancreatic head cancer patients. We looked at the three primary causes of this disease: (1) anorexia caused by insufficient pancreatic exocrine function; (2) difficulty in eating caused by mechanical compression; (3) increased consumption caused by systemic inflammatory response and tumor progression. Most studies have concluded that malnourished patients are more likely to have postoperative problems, and we have reached similar conclusions (5). Poor nutritional status has been linked to more severe postoperative sequelae and inflammation in previous research, but there was no statistical difference in the number of Clavien-Dindo grade (≥III) in our study. However, the lower PNI group had significantly longer hospital stays and higher surgical expenditures, indicating a slower postoperative recovery. Therefore, we concluded that with the improvement of surgical level and perioperative management, postoperative complications of patients were mostly Clavien-Dindo grade <III.

Recently, the preoperative LMR, NLR and PLR has been extensively studied in pancreatic cancer (6, 8, 18–22, 24, 25, 29–31). They discovered that these systemic inflammatory indicators can be employed as prognostic predictors of survival, which is in line with the findings of this study. PNI is also a predictor of OS along with these markers of systemic inflammation. In addition to this, we found that PNI has a strong linear correlation with LMR, NLR and PLR, but the mechanism needs to be further explored by basic research. Shinji et al. discovered that carbohydrate antigen 19-9 (CA19-9) was linked to poor clinical outcomes in patients with surgically resected pancreatic ductal adenocarcinoma (PDAC) (21). CA19-9 was also discovered to be an independent invasive and malignant predictor of intraductal papillary mucinous neoplasm(IPMN) by Shuji et al. (32). However, our data showed that carbohydrate antigen 125 (CA125) and CA19-9 did not show statistical differences in either univariate or multivariate analyses. This may be because our study included various pathological types of pancreatic head cancer rather than focusing on a specific pathological type. Some studies have shown that nerve invasion, vascular invasion and incisor involvement are important prognostic factors for patients with pancreatic cancer, but these parameters can only be obtained after surgery (33–35). Furthermore, in this investigation, there was no statistical difference in pathological parameters such as nerve infiltration, vascular infiltration and incisor involvement. PNI was revealed to be a predictor of survival for pancreatic head carcinoma in both univariate and multivariate analysis. It is important to note that PNI can be obtained by preoperative hematology, which is very simple and affordable procedure.

There are certain limitations to our research. For starters, because this is a short-term retrospective study, we don’t have enough data to verify our research conclusions. Second, whereas Glasgow and CRP-based prognostic assessment systems have been found to be helpful and important indicators of overall survival for pancreatic cancer, CRP wasn’t routinely evaluated in our patient population. Third, we were unable to confirm the association between prognostic variables and various types of postoperative complications due to a lack of data,. As a result, large-scale prospective studies must be established in order to verify conclusions and collect more clinical data to comprehensively evaluate the effects of various predictive indicators. Iannone et al. also pointed out that the operation strategy influence on patients’ immune response, considering the relationship of PNI and immune response markers in this study, we think low PNI can affect patients with surgical strategies, but there is no strong enough evidence in the world to support our operations strategy changes, so as our center of diagnosis and treatment strategy, total mesopancras excision (TMpE) was performed in all patients. Further studies on PNI and surgical strategies are expected (36).

In conclusion, our findings show that PNI is linked to a variety systemic inflammatory response marks and can be used as an independent prognostic factor in patients with pancreatic head cancer.

## Data Availability

The raw data supporting the conclusions of this article will be made available by the authors, without undue reservation.
